# Separase Loss of Function Cooperates with the Loss of p53 in the Initiation and Progression of T- and B-Cell Lymphoma, Leukemia and Aneuploidy in Mice

**DOI:** 10.1371/journal.pone.0022167

**Published:** 2011-07-25

**Authors:** Malini Mukherjee, Gouqing Ge, Nenggang Zhang, Eryong Huang, Lanelle V. Nakamura, Marissa Minor, Viacheslav Fofanov, Pullivarthi H. Rao, Alan Herron, Debananda Pati

**Affiliations:** 1 Department of Pediatric Hematology/Oncology, Texas Children's Cancer Center, Baylor College of Medicine, Houston, Texas, United States of America; 2 Department of Pathology and Immunology and Center for Comparative Medicine, Baylor College of Medicine, Houston, Texas, United States of America; 3 Eureka Genomics®, Corp, Houston, Texas, United States of America; Texas A&M University, United States of America

## Abstract

**Background:**

Cohesin protease Separase plays a key role in faithful segregation of sister chromatids by cleaving the cohesin complex at the metaphase to anaphase transition. Homozygous deletion of *ESPL1* gene that encodes Separase protein results in embryonic lethality in mice and Separase overexpression lead to aneuploidy and tumorigenesis. However, the effect of Separase haploinsufficiency has not been thoroughly investigated.

**Methodology/Principal Findings:**

Here we examined the effect of *ESPL1* heterozygosity using a hypomorphic mouse model that has reduced germline Separase activity. We report that while *ESPL1* mutant (*ESPL1 ^+/hyp^*) mice have a normal phenotype, in the absence of p53, these mice develop spontaneous T- and B-cell lymphomas, and leukemia with a significantly shortened latency as compared to p53 null mice. The *ESPL1* hypomorphic, p53 heterozygous transgenic mice (*ESPL1* ^+/hyp^, *p53^+/−^*) also show a significantly reduced life span with an altered tumor spectrum of carcinomas and sarcomas compared to p53^+/*−*^ mice alone. Furthermore, *ESPL1^+/hyp^, p53^−/−^* mice display significantly higher levels of genetic instability and aneuploidy in normal cells, as indicated by the abnormal metaphase counts and SKY analysis of primary splenocytes.

**Conclusions/Significance:**

Our results indicate that reduced levels of Separase act synergistically with loss of p53 in the initiation and progression of B- and T- cell lymphomas, which is aided by increased chromosomal missegregation and accumulation of genomic instability. *ESPL1* ^+/hyp^, *p53^−/−^* mice provide a new animal model for mechanistic study of aggressive lymphoma and also for preclinical evaluation of new agents for its therapy.

## Introduction

An evolutionarily conserved complex of proteins called cohesin is responsible for holding sister chromatids together prior to anaphase to prevent premature separation of sister chromosomes [1], [2]. At the onset of anaphase, cohesin subunit Rad21 is cleaved by the endopeptidase Separase which then allows sister chromatid separation and normal cell division to progress. Early cleavage of Rad21 by Separase can lead to abnormal accumulation of chromosomes and this is prevented by the tight regulation of Separase by its inhibitory chaperone Securin [3]–[6]. Phosphorylation of Securin by the Anaphase Promoting Complex/Cyclosome (APC/C) results in its ubiquitin-mediated degradation and release of free Separase which then cleaves cohesin Rad21 [7]–[11]. Phosphorylation of Separase by Cyclin B/Cdk1 also negatively regulates its activity and prevents premature Rad21 cleavage [12], [13]. It is therefore not surprising that the premature cleavage of the cohesin complex by early activation of Separase or by increased Separase activity results in the accumulation of abnormal chromosome number leading to aneuploidy. For example, increased expression of Separase in the mouse mammary epithelium results in the rapid accumulation of aneuploidy resulting in subsequent tumorigenesis [14], [15]. Also, overexpression of Separase has been detected in several human tumors, including breast and prostate tumors and osteosarcoma [16] suggesting a direct or indirect role of Separase in the etiology of human cancers. Furthermore, loss of catalytic activity of Separase caused by a truncating mutation in the gene has been shown to cause increased epithelial tumors along with a significant increase in genetic instability in a zebra fish model [17]. Correlative studies have reported the loss or decrease in Separase expression levels caused by somatic mutations in the *ESPL1* gene in human lung and kidney cancers (Catalogue of Somatic Mutations in Cancer, COSMIC database; [18]). Despite implications from the above mentioned studies that loss of Separase may have a role in the initiation and/or progression of cancer, the direct effects of loss of Separase if any on carcinogenesis have not been well studied.

SiRNA mediated knockdown of Separase in HeLa cells and Separase deficient mouse embryonic fibroblasts results in polyploidy [10], [19]. Separase knockout mice are embryonic lethal [20], [21] and no significant phenotype in Separase heterozygous mice have been reported to date. However, given the critical role of Separase in chromosomal segregation, it is likely that either the loss or gain of functional mutations of *ESPL1* can have serious consequences and may also lead to tumorigenesis.

Human cancer progresses as a multi-step process with cooperation between loss of key tumor suppressor genes as well as activation of oncogenes [22], [23]. Therefore, looking at the combined effect of losses or gains in several genes to see if that promotes or accelerates tumorigenesis has been a well accepted strategy in the development of mouse models of human cancer. p53 is one of the most frequently used candidates that have been shown to collaborate with other genetic mutations to promote initiation and progression of several malignancies [24]–[28]. Loss of p53 is an extremely frequent event in many human solid tumors and is also detected fairly often in hematopoietic tumors (http://www-p53.iarc.fr/GraphSTAT.asp?TypeGraph=TumorPrevalence). Individuals born with a single mutated copy of p53 have the cancer pre-disposing Li-Fraumeni syndrome that pre-disposes them to the development of many different cancers in their life time [29], [30].

Many mouse models have been established over the past several years to study the role of gain or loss of p53 function in various cell types [31]–[34]. These studies showed that mice homozygous for p53 develop a variety of spontaneous neoplasms and predominantly thymic lymphoblastic lymphomas with latency from four to six months, depending on the genetic background of the mice. Mice heterozygous for p53 also develop a variety of neoplasms, specifically osteosarcomas and soft tissue carcinomas, although with a longer latency of eight months and more [31]. Tumor latencies in these mice are also greatly influenced by the strain background. These results suggest that loss of p53 is an event that allows other stochastic mutations to accumulate within the cell resulting in cancer formation. So rather than being a “causal” event, loss of p53 is an “enabling” event for cancer initiation and progression. Given the already established premises that loss of p53 leads to a reduction in apoptosis and DNA damage repair mechanisms, and also the putative role of Separase in chromosomal segregation [35] and DNA damage repair pathways [19], [36], it was our hypothesis that loss of function mutations of both of these genes can collaborate in initiating and promoting malignant transformation in multiple cell types.

In an effort to understand the consequences of reduction of Separase levels *in vivo* both alone and in cooperation with p53 loss, we have developed and characterized Separase hypomorphic mice (referred to here as *ESPL1^+/hyp^* mice) with reduced Separase protein level, in the absence and presence of p53. We report here that mice heterozygous for the Separase hypomorph are viable in a C57Bl/6J background but the compound Separase hypomorphic, p53 homozygous mice (*ESPL1^+/hyp^*, *p53^−^* ^/*−*^) in the same genetic background have a significantly increased rate of tumorigenesis, shortened life span and mortality reaching 100% within 4–6 months of life. *ESPL1^+/hyp^, p53^−/−^*mice develop aggressive mixed T- and B-cell lymphomas along with leukemia involving the blood and bone marrow. Interestingly, normal tissues in these mice display significantly increased aneuploidy and chromosomal aberrations suggesting aneuploidy as precursor of tumorigenesis. These findings collectively indicate that reduction in Separase levels cooperate with loss of p53 in the generation of aggressive T- and B- cell lymphomas and leukemia in mice through increased aneuploidy and accumulation of multiple chromosomal aberrations.

## Results

### 1. Generation of Separase hypomorphic mice

**Figure 1:**
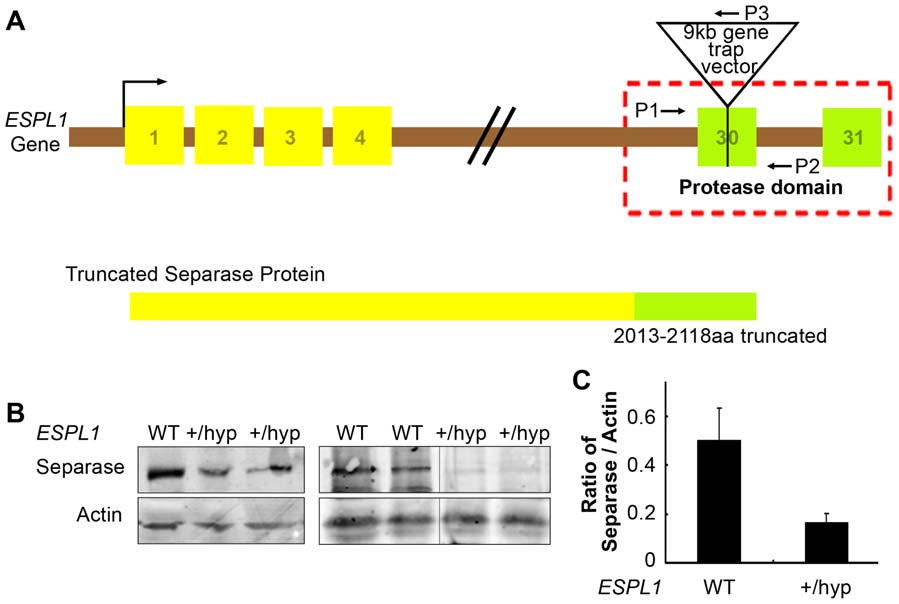
Disruption of *ESPL1* gene that encodes Separase protein (*A*) leads to the disruption of the C-terminal peptidase domain of the Separase protein. *Top*, schematic of *ESPL1* gene mutation by insertion of a gene trap vector, resulting in a truncated Separase protein is shown. Primer sets P1 and P3 are used to genotype the mice with mutant allele and P1 and P2 are used to genotype mice with the wild type allele. (***B*** *)*, Western blot analysis of endogenous Separase protein from testis derived from the mutant mice and their wild type littermate controls (as indicated by the genotypes). *(* ***C*** *)*, Densitometric quantification of Separase expression in the *ESPL1^+/hyp^* mice compared to wild type after normalization to the expression of a housekeeping gene β-actin to compensate for loading control.

To evaluate the effects of reduced expression of Separase, Separase hypomorphic mice were generated in a C57BL/6 background using the Baygenomics genetrap clone XL058. In this clone the gene trap insertion selectively disrupts the last but one exon (exon 30) of the *ESPL1* gene resulting in effective disruption of the C-terminal 105 amino acids encoding a major part of the peptidase domain of the Separase protein (Figure 1, A). Homozygous XL058 *ESPL1* mutants were embryonic lethal at E6.5 days, confirming the previous report for an essential role of Separase [20], [21]. *ESPL1^+/hyp^* mice were born normally with no apparent abnormality or disease phenotype over two years of observations. Due to lack of a suitable C-terminal Separase antibody, Separase expression in the hypomorphic mice was carried out using an N-terminal antibody. Analysis of Separase expression in testis and other tissues (mammary gland and ovary, data not shown) using immunoblot analysis indicated a reduction in the total Separase protein level in the *ESPL1^+/hyp^* mice, compared to wild type littermate controls (Figure 1B), indicating a possible instability of the truncated C-terminal protein. It is also interesting to note that Separase transcript level in the *ESPL1^+/hyp^* mice was found to be slightly higher than that in *WT* mice (data not shown) again suggesting that an unstable Separase protein results from insertion of the gene cassette in the hypomorphic mice, resulting in decrease in protein but not in mRNA levels.

### 2. Reduction in Separase levels cooperates with loss of p53 in the formation of T and B cell lymphomas in mice

**Figure 2:**
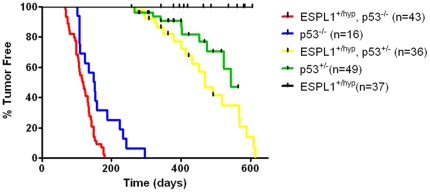
Lymphoma onset and progression are accelerated by Separase reduction. Double mutant (*ESPL1^+/hyp^,p53^−/−^*) animals developed severe lymphomas with a median latency of 118 days and within 200 days all animals developed full blown lymphomas (red line). In *p53^−/−^* animals (blue line), lymphomas were detected significantly later (*p*<0.0001) with a median onset of 151 days and progression of lymphomas was significantly slower, measured by sick appearance of the mice and subsequent histopathological analysis. The *ESPL1^+/hyp^, p53^+/−^* mice (yellow line) do not develop lymphomas but develop other phenotypes over a longer period of time.

To examine if a reduction in Separase levels leads to chromosome missegregation and increases the risk of tumorigenesis, survival analysis was performed using littermate cohorts of Separase heterozygous hypomorphic mice (*ESPL1^+/hyp^*) and control WT mice in identical C57Bl/6 genetic backgrounds. Animals were sacrificed when terminally ill or when they reached the age of 24 months (∼2 years). Animals sacrificed at the study end point were censored in survival data if they did not display tumor phenotype. However, upon observation of mice maintained in our colony beyond the time period shown in survival curve (Figure 2), we found that the *ESPL1^+/hyp^* animals had a longer mean average tumor-free lifespan (more than 800 days vs. 600 days for WT) compared to that reported for WT C57Bl/6 animals [37]. As the functional loss of a single allele of *ESPL1* occurred in all tissues, mice were examined for all possible tumor types upon necropsy. We failed to observe any tumors or any other obvious abnormalities in the *ESPL1^+/hyp^* mice during their life span though about 5% of the control cohort of littermate WT mice developed sarcomas and appeared sick (possible lymphomas) and had to be sacrificed within a 2 year time frame.

Since the *in vivo* effects of mitotic checkpoint impairment and chromosome missegregation are not very well defined, we took into consideration the possibility that cell cycle checkpoints turned on by the presence of an active p53 protein might override tumorigenic effects of the loss of a single allele of *ESPL1*. That is, cells lacking Separase that develop chromosomal abnormalities may be eliminated by p53-dependent checkpoint arrest or apoptosis. If so, the introduction of p53 mutations in *ESPL1^+/hyp^* animals would be expected to cooperate with reduction of Separase protein levels in tumorigenesis. Additionally, if loss of Separase expression increases the rate of chromosomal missegregation *in vivo*, *ESPL1^+/hyp^* mice on a p53 heterozygous background should be more likely to experience loss of the wild-type p53 allele to allow tumors to progress. Loss of heterozygosity of p53 has been suggested as a possible mechanism and also a rate limiting step in the development of tumors upon irradiation in p53 heterozygous mice [38]. To test these hypotheses, survival analysis was performed on cohorts of *ESPL1^+/hyp^* animals and littermate controls generated on *p53* heterozygous and homozygous backgrounds. *ESPL1^+/hyp^, p53^−/−^* mice developed aggressive and widespread lymphomas involving the lung, liver, thymus, bone marrow and peripheral blood, with a significantly reduced latency compared to *p53^−/−^* animals alone (Figure 2, red vs. blue line, P<0.005, Log Rank Test). Also the *ESPL1^+/hyp^, p53^+/−^* mice developed carcinomas in various organs, with a significantly reduced latency compared to the *p53* heterozygous mice alone (P<0.005, Log Rank Test) in a C57BL/6 background. Approximately 86% of the *ESPL1^+/hyp^, p53^−/−^* animals developed lymphomas with a median survival period of 118 days, compared to about 37% *p53^−/−^* animals that developed pathologically confirmed lymphomas in the same time period. On the other hand, approximately 50% compound heterozygote (*ESPL1^+/hyp^, p53^+/−^*) animals developed carcinomas over a period of 460 days (about 14 months), compared to *p53^+/−^* animals, a much smaller percentage of which developed a significant tumor phenotype over the same time period. Together, these data suggest that reduced levels of Separase co-operates with the loss of p53 in the initiation and progression of tumors, with other stochastic changes occurring along the way to initiate tumor formation. The *ESPL1^+/hyp^, p53^+/−^* and *ESPL1^+/hyp^*, *p53^+/+^* mice do not develop lymphomas over a period of 200 days (pre-dominant phenotype in each genotype summarized in Table 1).

**Table 1:** Tumor incidences and morphologies displayed by various *ESPL1* and *p53* genotypes.

Tumor Type (%)	Genotype				
	*ESPL1^+/hyp^, p53^−/−^*	*p53^−/−^*	*ESPL1^+/hyp^*, *p53^+/−^*	*ESPL1^+/hyp^*	*p53^+/−^*
Lymphoma	100	70	0	0	0
Carcinoma	9	0	40	0	0
Sarcoma	0	50	10	0	0
**Lymphoma Infiltration (%)**					
Thymus	100	100	0	0	0
Liver	20–80	0	0	0	0
Spleen	80	25	0	0	0
Lung	20–50	5	0	0	0
Kidney	0	0	0	0	0
Colon	2	0	0	0	0
Bone Marrow	30	0	0	0	0
Peripheral Blood	40	0	0	0	0

*Since mice from certain genotypes have multiple tumor types and show lymphoma infiltration in multiple organs, the sum exceeds 100%*.

### 3. Reduction in Separase levels contributes to increased aggressiveness and metastatic profile of lymphomas in the *ESPL1^+/hyp^, p53^−/−^* mice

**Figure 3:**
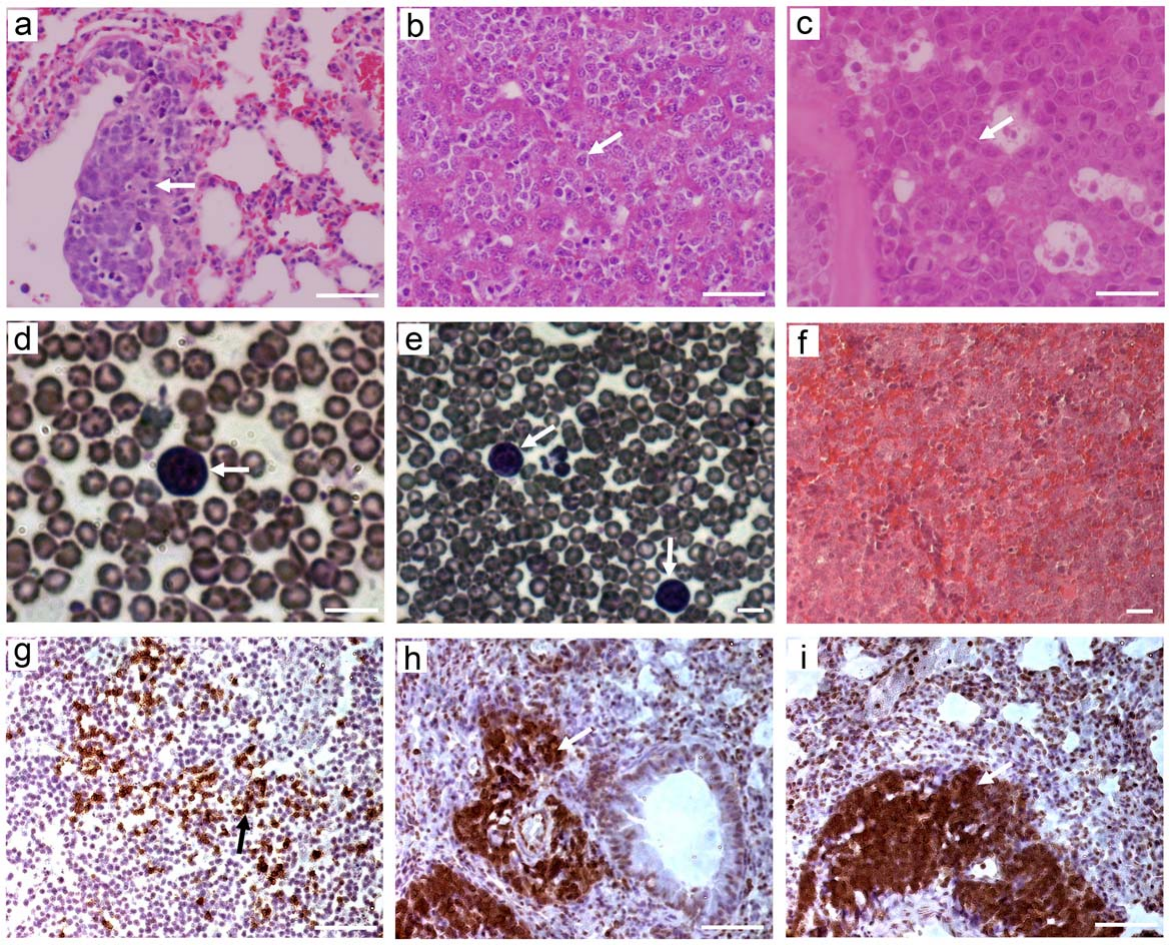
*ESPL1^+/hyp^, p53^−/−^* mice develop widely disseminated lymphoma and leukemia involving the lung, liver, spleen, bone marrow and peripheral blood. Hemotoxylin and eosin staining of *ESPL1^+/hyp^, p53^−/−^* mice lymphoma affected tissue sections shows that the terminal stage lymphomas have varied morphologies. Poorly differentiated and homogenous populations of atypical round lymphocyte cells were observed in the lung alveoli (**a, arrow**) showing acute lymphoma invasion in the lungs. The liver tissues in these mice (**b**) were depleted by atypical lymphocytes and homogenous round cells with frequent large pleomorphic nuclei infiltrating between the hepatic cores and sinusoids (**b, arrow**). Invasion of lymphoma was observed in the bone marrow (**c, arrow**) and presence of blast cells suggested leukemic infiltration in the peripheral blood (**d, e**, **arrows**). Frequent invasion of lymphoma was also observed in the spleen (**f**) resulting in complete loss of normal spleen morphology. Both B cell positive (**g**, brown stain, arrow, B220 positive) and T cell positive (**h** and **i**, brown stain, arrow, CD3 positive) lymphoma cells were observed in these lymphomas. Percentages of tumor types displayed in the various genotypes are tabulated in Table 1. Scale bar = 50 µm.

Homozygous p53 null mice (*p53^−/−^*) have been reported to develop predominantly T cell thymic lymphomas with infrequent involvement of lymph nodes in multiple organs including liver, lung and kidney. Crossing the *p53^−/−^* mice to the *ESPL1^+/hyp^* mice to generate the *ESPL1^+/hyp^, p53^−/−^* mice significantly altered the lymphoma profile as well as the latency for lymphoma development. The double mutant mice developed mixed T and B cell lymphomas (Figure 3g–i) shown by staining for T and B cell specific markers B220 and CD3, respectively). In contrast to the *p53^−/−^* mice, lymphoma in *ESPL1^+/hyp^, p53^−/−^* were seldom localized to the thymus, but most commonly infiltrated to the lungs, liver and spleen. The lungs (Figure 3a), liver (Figure 3b) and spleen (Figure 3f) in these mice were often completely depleted by lymphoma cells and normal anatomy of these organs was not visible. In all these organs very high levels of proliferation was observed coupled with the presence of homogenous sheets of atypical round cells and large pleomorphic nuclei. Invasion of lymphoma cells was also observed less frequently in the colon and kidney and on the cardiac surface in these mice (data not shown). Furthermore, the double mutant mice had significant lymphoma invasion in the bone marrow (Figure 3c) and in the peripheral blood, as seen by the appearance of blasts in blood smears from these mice (Figure 3d, 3e, arrow), indicating that they had a leukemic profile. The percentage of animals displaying each of these phenotypes is summarized in Table 1. These findings suggest that the reduction in Separase in the absence of p53 plays a synergistic role in contributing to the severe phenotype in the hematopoietic system.

### 4. Lymphomas in *ESPL1^+/hyp^, p53^−/−^* mice have increased levels of proliferation and DNA damage

**Figure 4:**
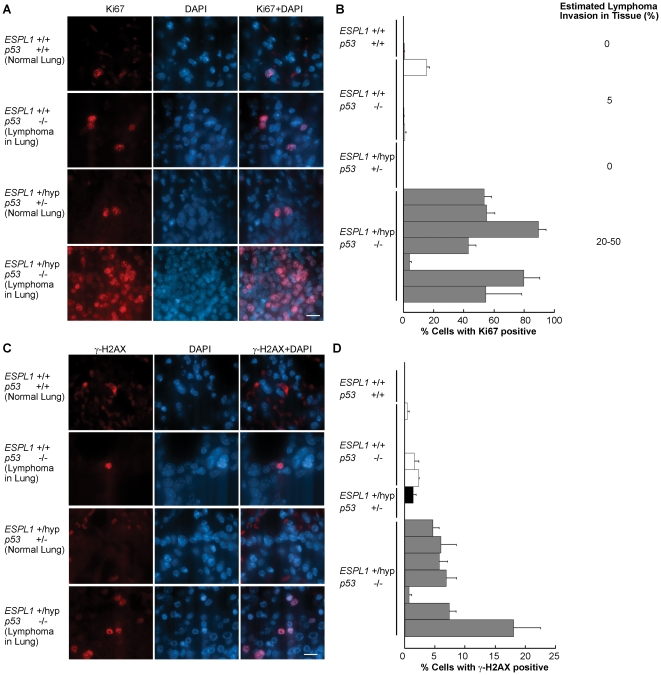
*ESPL1^+/hyp^, p53^−/−^* mice show increased proliferation and DNA damage in the lungs. Lungs from WT, *p53^−/−^*, *ESPL1^+/hyp^, p53^+/−^* and *ESPL1^+/hyp^, p53^−/−^* mice were analyzed for cellular proliferation. Only lung tissue from *p53^−/−^* and *ESPL1^+/hyp^, p53^−/−^* mice showed lymphoma invasion and lung tissue from WT and *ESPL1^+/hyp^, p53^+/−^* were normal. Proliferation levels are indicated by the percentage of cells in any cycling phase of the cell cycle (G1, S, G2, M) (**B**) calculated by counting Ki67 cells (red) as a percentage of total cells (DAPI, blue) as shown in (**A**) (n = at least two mice for each genotype were used and for each genotype 5 microscope fields and>500 cells were counted.). Each bar represents an individual mouse for that genotype. Significant difference was observed between proliferation levels in the lymphoma invaded lungs of the *ESPL1^+/hyp^; p53^−/−^* mice compared to all other cohorts (*P = 0.01*). Estimated percentage lymphoma invasion in lungs of mice from each genotype is shown in B. Lungs from WT, *p53^−/−^*, *ESPL1^+/hyp^, p53^+/−^* and *ESPL1^+/hyp^, p53^−/−^* mice were analyzed for DNA damage. DNA damage levels are indicated by the percentage of cells stained positive for γH2AX (**D**) calculated by counting cells showing detectable levels of γH2AX foci (red) as a percentage of total cells (DAPI, blue) as shown in (**C**). Significant difference was observed between DNA damage foci levels in the *ESPL1^+/hyp^*, *p53^−/−^* mice compared to all other cohorts (*P = 0.005*). Scale bar = 20 µm.

As Separase plays a critical role in the ability of cells to complete mitosis and go through cell cycle, we checked the levels of proliferation in the tissues affected by lymphoma (identified by histological examination) of the *ESPL1^+/hyp^* mice in various *p53* backgrounds. To carry out these experiments, tissue sections from lymphoma invaded lung, liver and thymus in the terminal mice were stained with the proliferation marker Ki67. We observed that while normal lungs from wild type age matched litter mate mice exhibited very low levels of proliferation (exhibited by Ki67 positive staining of cycling cells), the *ESPL1^+/hyp^ p53^−/−^* mice showed a striking increase in proliferation in their lung tissue with up to 80% cells stained positive for Ki67 (Figure 4A and 4B). The lymphoma invasion in the lungs in these mice ranged from 20–50% (as seen by H&E observation of lung sections, Figure 3A), indicating that possibly both lymphoma as well as lung epithelial cells contributed to the increase in proliferation. Lung tissue (Figure 4A, second panel from top) and thymus tissue sections (data not shown) taken from the *p53^−/−^* mice that showed lymphoma invasion in lung and thymus did not show a significant increase in proliferation above control levels, suggesting that the cells contributing to lymphomagenesis in the double mutant mice had a proliferative advantage that could contribute to the decreased latency in their lymphoma progression.

To further identify causes for the shortened latency and more aggressive lymphoma development in the double mutant mice and also to investigate the possible contribution of Separase *in vivo* in DNA damage repair [39]–[46] we examined the level of DNA damage in various lymphoma affected tissues of these mice. DNA damage was assayed by staining tissue sections with gamma-H2AX, a commonly used DNA damage marker and counting cells stained positive for this marker. We observed that while wild type age matched littermate mice had very few gamma H2AX positive cells in their lungs (due to absence of lymphoma cells), the double mutant mice had a significantly high percentage of cells that stained positive for gamma-H2AX (Figure 4C and 4D). Up to a 15% increase in DNA damage was detected in the lymphoma invaded lungs of the double mutant mice. The *p53^−/−^* mice did not show a significant rise in DNA damage in their lymphoma affected tissues compared to the wild type litter mate cohorts.

To further investigate the levels of proliferation and DNA damage accumulation in pre-neoplastic (not affected by lymphoma) tissues in these mice, primary splenocytes and bone marrow cells from *ESPL1^+/hyp^, p53^−/−^, p53^−/−^ and WT* mice at the average age of three months (not affected by lymphoma) were stained for Ki67 and gamma H2AX markers. The normal (potentially pre-neoplastic splenocytes and bone marrow cells obtained from the double mutant (*ESPL1^+/hyp^, p53^−/−^*) showed significantly higher levels of proliferation compared to the *p53^−/−^* and WT cohorts (Figure S1). However any significant increase in DNA damage was not observed in any genotype of these primary bone marrow and splenocytes (data not shown).

### 5. Aberrant nuclear localization of Separase in Interphase nuclei of lymphomas co-localizes with DNA damage foci

**Figure 5:**
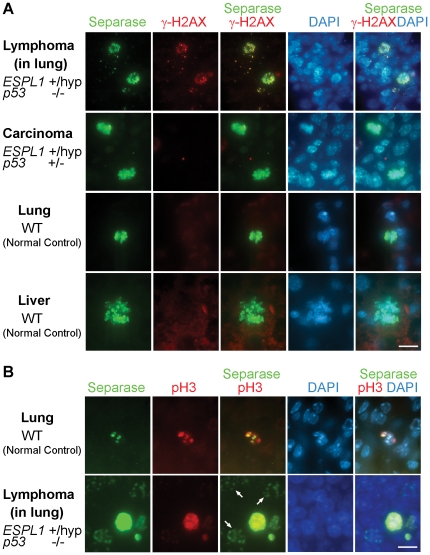
Lymphomas in *ESPL1^+/hyp^, p53^−/^−* mice have increased nuclear localization of Separase protein that co-localizes with DNA damage foci. Tumor sections from *ESPL1^+/hyp^, p53^−/−^* (A, lung invaded with lymphoma, **top panel** *)* and *ESPL1^+/hyp^, p53^+/−^* (A, colon carcinoma, **middle panel**) and WT (A, normal lung, third **panel,** normal liver**, bottom panel**) mice were co-stained with Separase and gamma H2AX antibodies. A significant increase in nuclear co-localization of Separase and gamma H2AX was observed in the lymphoma tissue from the *ESPL1^+/hyp^*, *p53^−/−^* mice compared to matched normal tissues and carcinomas taken from the *ESPL1^+/hyp^, p53^+/−^* and *p53^−/−^* mice. Carcinomas from the *ESPL1^+/hyp^*, *p53^+/−^* showed insignificant levels of gamma H2AX staining comparable to wild type tissue and nuclear Separase was only seen in mitotic cells in these genotypes. (Separase = Green, γH2AX = Red). Separase localization in interphase nuclei (non-mitotic cells) was observed in the lymphoma invaded lung tissue when co-stained with a mitotic cell marker (anti- phospho histone H3 (Ser 10), Panel B, bottom). In normal lung, co-localization of Separase and PH3 was only seen in mitotic cells (Panel B, top). (Separase = Green, PH3 = Red). Scale bar = 10 µm.

As Separase has been implicated in DNA damage repair pathways [19] we checked the levels of Separase protein in the tumor tissues (identified by histological examination) of the *ESPL1^+/hyp^* mice in various p53 backgrounds. To carryout these experiments, tissue sections from lymphoma invaded lung, liver and thymus in the terminal *ESPL1^+/hyp^, p53^−/−^* mice as well as tissues affected by various carcinomas (colon, mammary and salivary) in the *ESPL1^+/hyp,^ p53^+/−^* mice were stained with Separase antibody. We observed that while various tissue sections from normal wild type age matched litter mate mice exhibited very low levels of Separase protein (seen only in mitotic cells, wild type lung and liver, Figure 5A, bottom two panels), the *ESPL1^+/hyp^, p53^−/−^* mice showed a striking increase in interphase nuclear Separase levels in the lymphoma invaded tissue (Figure 5A, top panel). Tissue sections taken from the carcinomas in *ESPL1^+/hyp^, p53^+/−^* mice failed to show similar increased nuclear Separase levels in interphase cells and were comparable to wild type mouse tissue (Figure 5A, colon carcinoma, second panel). Furthermore, significant co-localization of the nuclear Separase staining in the lymphomas from *ESPL1^+/hyp^, p53^−/−^* mice was observed with the DNA damage marker gamma H2AX (Figure 5A, top panel). The carcinomas and wild type tissue sections had no observable DNA damage, and Separase staining was only observed in mitotic cells in these tissues (Figure 5A, bottom two panels). To ascertain that Separase was localized to the nuclei of non-mitotic (interphase) cells in the lymphomas derived from the *ESPL1^+/hyp^, p53^−/−^* mice, co-staining was performed with Separase antibody and a anti-Phospho Histone H3 (Ser 10) antibody as a mitotic marker (Figure 5B, bottom panel). A fraction of cells in the lymphoma invaded lungs were found to stain for Separase alone and not for anti-PH3, whereas in normal lung tissue, Separase staining was only observed in anti-PH3 positive (mitotic) cells (Figure 5B, top panel).

### 6. Reduction in levels of p53 and Separase co-operate in tumor progression

**Figure 6:**
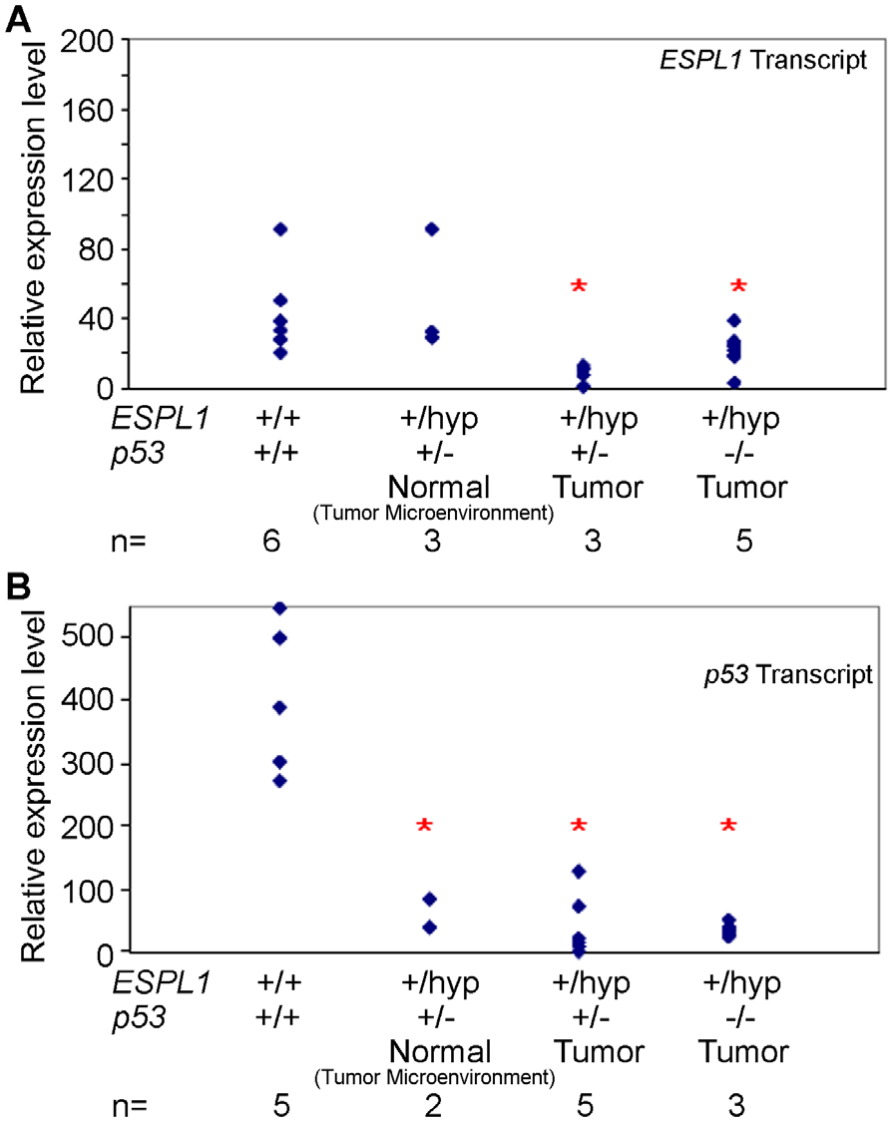
Q-PCR analysis of lymphomas and carcinomas arising in the *ESPL1^+/hyp^* mice in varying *p53* backgrounds reveals significant reduction of p53 and Separase mRNA levels. Separase and p53 transcripts in various tumor tissues was compared across different mouse genotypes to matched wild type litter mate mouse tissue. (**A**) A comparison of the relative Separase mRNA expression levels in wild type and *ESPL1* mutant mice in a C57BL/6 background indicates a significant reduction in Separase expression levels both in the *ESPL1^+/hyp^, p53 ^−/^* ^***−***^ lymphoma tissue (P = 0.0003) as well as in the carcinomas from the *ESPL1^+/hyp^*, *p53^+/−^* mice (P = 0.007). (**B**) Relative mRNA expression levels of p53 in wild type and mutant mice in a C57BL/6 background are shown. Tissue taken from both tumor microenvironment and tumor tissue from the same *ESPL1^+/hyp^, p53^+/−^* mice show a significant reduction in p53 transcript levels compared to levels of p53 in wild type mice tissue (P = 0.003 and P = 0.004 respectively). RNA isolated from *ESPL1^+/hyp^, p53 ^−/−^* mice lymphomas was used as a control for p53 transcript levels. (‘n’ denotes number of tumors and red star denotes significant difference compared to wild type tissue).

The protein level in the *ESPL1^+/hyp^, p53^+/−^* was comparable to the *ESPL1^+/hyp^* animals, but lower then WT animals (see Figure S2). To assess if there was a selective pressure for the loss of the wild type allele of *ESPL1* during lymphoma progression in mice, real-time quantitative PCR analysis was employed to determine the status of the wild-type *ESPL1* allele in *ESPL1^+/hyp^, p53^−/−^* and *ESPL1^+/hyp^, p53^+/−^* tumors. Our analysis showed a significant reduction in Separase transcript levels in carcinomas compared to matched normal tissues in the *ESPL1^+/hyp^, p53^+/−^* mice (Figure 6A). Reduction in Separase transcript levels was also observed in a fraction of the lymphomas from *ESPL1^+/hyp^, p53^−/−^* mice compared to wild type mouse tissue (Figure 6A). Furthermore, to assess the role of p53 in the progression of carcinomas in the *ESPL1^+/hyp^, p53^+/−^* mice, we analyzed tumor and matched normal tissue (taken from the area surrounding the tumor) from these mice (Figure 6B). A significant reduction in p53 transcript levels (compared to wild type) was noted in the tumor (P = 0.0003) as well as tissue surrounding the tumor (tumor microenvironment) (P = 0.0001) taken from the *ESPL1^+/hyp^ p53^+/−^* mice, suggesting that loss of p53 function in the tumor tissue itself as well as tumor microenvironment was a rate limiting step in the progression of these tumors. Tissue from *ESPL1^+/hyp^ p53^−/−^* mice was used as a control for p53 transcript levels.

### 7. Reduced Separase levels promote aneuploidy in *p53^−/−^* mice

**Figure 7:**
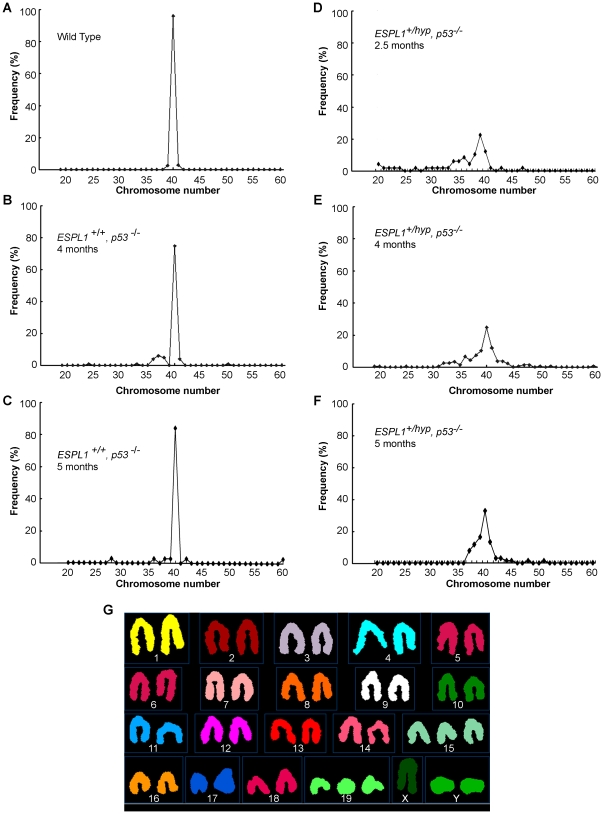
*ESPL1^+/hyp^, p53^−/−^* primary splenocytes display increased aneuploidy. Distribution of chromosome numbers in the three genotypes are shown here (**A**, Wild Type*;* **B**, **C** p53*^−^* ^/*−*^, **D, E, F**, *ESPL1^+/hyp^, p53^−/−^*). Average counts from splenocytes of two normal mice (tumor free) per genotype, (average age 4 months for wild type, 4 and 5 months for p53*^−/−^* mice and 2.5, 4 and 5 months for *ESPL1^+/hyp^, p53^−/−^* mice) was plotted. At least 50 metaphases were counted per genotype. A representative SKY image (**G**) of splenocytes of a 4 month old *ESPL1^+/hyp^, p53^−/−^* mouse shows gains in chromosomes 15, 19 and Y (the change in Y chromosome number seen here is not clonal).

To determine if altered Separase expression co-operates with loss of p53 in lymphoma progression by accumulating aneuploidy, we performed metaphase spread counts on splenocytes and bone marrow cells isolated from wild type, *p53^−/−^* and *ESPL1^+/hyp^, p53^−/−^* mice (Figure 7A–F). Primary splenocytes and bone marrow cells were sampled from normal tumor-free mice of all three genotypes, at multiple ages (time points) to detect pre-malignant changes in chromosome numbers if any. Metaphase spread counts revealed a significant increase in abnormal chromosome numbers (both gain and loss, tabulated in Table 2) in the double mutant mice compared to p53*^−^* ^/*−*^ mice alone. Accumulation of aneuploidy was detected in splenocytes in the double mutant (*ESPL1^+/hyp^, p53^−/−^*) mice as early as by 2.5 months of age (Figure 7D). Further analysis of normal (lymphoma free) splenocytes from four month old *ESPL1^+/hyp^, p53^−/−^* mice by SKY analysis revealed widespread and clonal chromosomal changes with a composite karyotype 36–70, XY, +3, +12, +14, +15, +19[cp6]/40, XY [15], showing gains in chromosomes 3, 12, 14, 15 and 19 (a representative example of some of the changes shown in Figure 7G). Similar chromosomal changes were not noted in splenocytes isolated from age matched *p53^−/−^* mice.

**Table 2:** Analysis of aneuploidy in normal (tumor free) splenocytes and bone marrow cultures.

Genotype	Tissue	% Aneuploidy
WT (1)	Spleen	0
	Bone Marrow	0
WT(2)	Spleen	2
	Bone Marrow	0
*p53^−/−^* (1)	Spleen	26
	Bone Marrow	Not available
*p53^−/−^ (*2)	Spleen	23
	Bone Marrow	Not available
*ESPL1^+/hyp^, p53^−/−^* (1)	Spleen	59
	Bone Marrow	81
*ESPL1^+/hyp^, p53^−/−^* (2)	Spleen	55
	Bone Marrow	88

Values are from two independent mice (average age = 4 months) for each genotype. At least 50 metaphase spreads were counted for each culture. (1 and 2 represent two individual mice).

## Discussion

Since its discovery in yeast as an important cell cycle component that holds sister chromatids together [47], [48], the cohesin proteins have been the focus of intense research. The role of loss of components of the cohesin complex in generating genetic instability [3], [49], DNA damage repair defects [39]–[46] as well as alterations in gene expression [50]–[53] have been studied in cell culture systems. Several studies have reported loss or gain of specific components of the cohesin complex, suggesting correlation between cancer initiation and/or progression and the expression and function of these proteins. Specifically recent evidence in multiple genetically unstable human cancers [54] have brought into speculation the possible role of cohesin protein defects in promoting aneuploidy that may play a causal role in tumorigenesis. However, in vivo models to study the effect of gain or loss of individual components of the cohesin complex in tumor development are currently lacking. Here we present evidence that reduction in cohesin protease, Separase in mice collaborates with p53 inactivation in the genesis and progression of aggressive B- and T-cell lymphoma and leukemia in mice. We show that the spectrum of lymphomas in these mice are completely different from those observed in p53 null mice alone and involve the spleen, lung and liver as well as bone marrow, but not the thymus. Our studies also demonstrate that lymphoma formation in the double mutant (*ESPL1^+/hyp^, p53^−/−^*) mice is greatly accelerated by genomic instability and aneuploidy that starts accumulating very early in these mice. We provide a mouse model for further understanding the biology and pathways disrupted in human lymphoma and leukemia.

### Separase hypomorphism co-operates with loss of p53 to promote a more aggressive lymphoma phenotype compared to p53 loss alone

Separase is an essential protein. Previous studies indicated that conditional depletion of Separase in mouse bone marrow causes aplasia and apparent death of hematopoietic cells other than erythrocytes [21]. Our model suggests that optimal levels of Separase acts as a tumor suppressor in the absence of p53 and that loss of Separase and p53 effectively synergize the lymphoma and leukemia formation. While loss of p53 alone results primarily in localized thymic T cell lymphomas, as has been reported before [31], the combined reduction of Separase with loss of p53 results in more widespread B- and T-Cell lymphoma and leukemia with gross involvement of the bone marrow. Direct synergy between p53 loss and Separase reduction is clearly demonstrated by the accelerated tumor development in *ESPL1^+/hyp^, p53^−/−^* mice compared to *p53^−/−^ (ESPL1 WT)* animals. Surprisingly, reduction in Separase levels alone does not lead to tumorigenesis, suggesting that very low levels of Separase within the cell may be sufficient to provide the enzymatic activity to cleave Rad21 and prevent chromosomal missegregation. Also, downstream anti-proliferative and pro-apoptotic effects of a functional p53 most likely prevent the accumulation of defects to lead to lymphomagenesis in these mice.

We show here that both the combined inactivation of Separase and p53 as well as a reduction in p53 gene dosage contributes to tumorigenesis. The reduced levels of Separase expression in only some but not all the lymphomas arising in the *ESPL1^+/hyp^*, *p53^−/−^* mice suggesting that in a tumor microenvironment, complete loss of Separase is a stochastic but non-essential event. Furthermore, spleen tissue from *ESPL1^+/hyp^, p53^−/−^* mice have high accumulation of genomic instability and aneuploidy as early as in 2.5 month old mice, indicative of the critical role of Separase in maintenance of genomic stability. In contrary, the double heterozygous mice (*ESPL1^+/hyp^*, *p53^+/−^*) develop tumors over a longer time, most likely resulting in the gradual accumulation of stochastic events. We have previously reported [14], [15] that over-expression of Separase results in the premature separation of sister chromatids leading to aneuploidy in the mammary epithelium, thus suggesting that optimal levels of Separase is required for the maintenance of a tumor free cellular milieu. We show here that Separase can potentially act as an oncogene or as a tumor suppressor, and its role depends on its level of expression and tissue type. Manipulation of Separase level may therefore, contribute towards the development of a novel therapy to target aneuploid tumors. Of interest is also the fact that the Separase mutant mice alone (*ESPL1^+/hyp^*) do not have a lymphoma phenotype. This may suggest that though loss of Separase is a predisposing condition for hematopoietic malignancy, the further progression of cancer is blocked by apoptotic and other cell death pathways induced by an active p53.

It is a well recognized concept now that more than one biological pathway needs to be deregulated in order to transform normal cells into malignant tumor cells [55]. Loss of p53 results in loss of apoptotic responses and cell cycle control. This is sufficient to develop thymic lymphomas that do not involve the bone marrow and are hence less aggressive in nature. Loss of Separase in the p53 null mice results in the accumulation of aneuploidy leading to the quickened progression of the disease as well as its heightened malignancy, involving widespread leukemia in the bone marrow.

### Combined loss of Separase and p53 promotes aneuploidy

*In vivo* studies in the mouse mammary epithelium have shown that over expression of Separase results in the development of gross aneuploidy suggesting an important aneuploidy promoting role of Separase [14], [56]. We show here that functional loss of single allele of *ESPL1* in the absence of *p53* is also sufficient to induce significant levels of aneuploidy in normal cells *in vivo*, as indicated by the abnormal metaphase counts and SKY analysis of primary splenocytes isolated from *ESPL1^+/hyp^, p53^−/−^* mice. The accumulation of these genetic abnormalities in these mice may also account for the increased DNA damage levels identified in the lymphomas that infiltrated the lungs and liver of these mice.

The accumulation of gross genomic instability as well as DNA damage in the absence of p53 induced apoptosis could be a mechanism that aids the quicker lymphomagenesis in the double mutant mice. Lymphocytes are rapidly proliferating cells that have a very quick turn over time and require extensive and efficient DNA damage repair. Separase has been implicated in having a role in DNA damage repair [19]. This is thought to happen by the Separase-mediated cleavage of Rad21 and the removal of the cohesin complex from the site of DNA double strand breaks, allowing repair factors to approach the DNA and facilitate repair. It has also been suggested that the release of Separase by Securin to allow cohesin cleavage during DNA damage repair is a p53 mediated process [57]. However, direct *in vivo* evidence of the role of Separase in DNA damage repair in higher eukaryotes is still lacking. We show here that reduced levels of Separase in the absence of p53 plays an important role in the accumulation of genomic instability in lymphocytes, possibly allowing the accumulation of gross chromosomal abnormalities as well as DNA damage finally resulting in acute lymphoma and leukemia development. Also important is the observation that *ESPL1^+/hyp^* mice alone in the presence of an active p53 do not develop lymphomas and also have a longer tumor-free life span compared to wild type mice, possibly suggesting that increased levels of apoptosis and p53 mediated downstream checkpoint pathways prevents the accumulation of genetic instability and DNA damage accumulation and prevent cancer progression in these mice.

### Loss of Separase causes a phenotypic change from “localized” thymic lymphoma to diffuse lymphoma and leukemia involving multiple organs, peripheral blood and bone marrow

Human lymphoma and leukemia have various subtypes indicating the involvement of many different cell lineages in its initiation and progression. In our model, lymphomas that developed in the *p53^−^* ^/*−*^ mice alone were restricted to the thymus with occasional involvement of the lymph nodes in the lung and liver. Histopathological analysis indicated no bone marrow involvement in these animals. However, the combined reduction of Separase levels with loss of p53 changed the lymphoma spectrum in these mice to a more widely disseminated phenotype, involving lymph nodes of the lung, liver, thymus and spleen and also showing gross bone marrow involvement. It is conceivable that reduction of Separase levels along with loss of p53 results in the accumulation of DNA damage and aneuploidy in an independent subset of hematopoietic cells (from those affected by loss of p53 alone), resulting in the appearance of a more widespread T- and B-cell lymphoma phenotype as well as bone marrow involvement. Understanding of the specific hematopoietic subsets in this model will require further investigation with the use of cell lineage specific markers.

In summary, *ESPL1^+/hyp^, p53^−/−^* mice provide a new animal model for aggressive lymphoma not only to study the mechanism of lymphoma development but also for preclinical evaluation of new agents for lymphoma therapy.

## Materials and Methods

### Transgenic animals

*mESPL1* gene-trapped ES cell clone (XL058) was obtained from Bay Genomics (San Francisco, CA). The gene trap is produced by a splice-acceptor-β-geo cassette within *Espl1* exon 30 deleting the C-terminal 105 amino acid, a critical portion of its peptidase domain. The ES cells were injected into 129/SV blastocysts, and resulting chimeras were crossed to C57Bl/6J females and screened for germline transmission by PCR. Animals used in experiments reported here were backcrossed at least ten generations to the C57Bl/6J background. The p53 mice were obtained from Jackson Labs in a C57BL6 strain. All animal experiments were approved by the Institutional Animal Care and Use Committee (IACUC) of the Baylor College of Medicine. All animal studies were carried out in strict compliance with federal and local guidelines for animal care and use.

### Immunoblotting

Mouse tissues were flash frozen in liquid nitrogen and stored at −80°C. Proteins were extracted in lysis buffer and quantified and Western Blot analysis performed as reported before [14]. Membranes were probed with anti mouse Separase monoclonal antibody (Abnova, Taipei, Taiwan; 1∶500), raised against the N terminal domain of Separase. The specificity of this antibody on mouse Separase was tested before by [16]. Equal loading was verified using a mouse monoclonal anti-ß-actin antibody (Sigma, St. Louis, MO).

### Survival and tumorigenesis analysis

Animals were monitored regularly for signs of illness up to the age of 2 years. Overall and tumor-free survival times and *P* values were determined by Kaplan-Meier and Wilcoxon's log rank analyses (*P* values are shown for comparison). Animals that were tumor free at necropsy were censored in the survival analyses. Animals that died of unknown causes were also censored in lymphoma-free survival analysis but not in overall survival analyses. Lymphoma diagnoses were based on histological confirmation of tumors. Comparison of tumor frequencies was done by Fisher's exact test with one-tailed *P* values. All statistical analyses were performed with Graph Pad Prizm software (GraphPad Software, La Jolla, CA).

### DNA damage assay

DNA damage assays were performed using specific anti mouse- H2AX (Millipore) and anti-rabbit Phospho Serine H2AX antibodies (Cell Signaling Technologies, Danvers, MA). Mitotic marker anti-Phospho Histone H3 (Ser 10) was used (1∶1000, Millipore, Catalogue # 06-570) to detect mitotic cells.

### Immunohistochemistry, metaphase spread and splenocytes and bone marrow cultures

All IHC and IF analysis was performed using previously published methods [16]. Metaphase spread from splenocytes and bone marrow cultures was performed based on previously reported techniques in [58].

### Quantitative PCR

Total RNA was extracted from mouse tissues by grinding the tissues in liquid nitrogen followed by Trizol (Invitrogen, Carlsbad, CA) RNA extraction. cDNA was synthesized by reverse transcription of mRNA using oligo-dT primers (Invitrogen, Carlsbad, CA) and Superscript II reverse transcriptase (Invitrogen) as instructed by the manufacturer. Quantitative real-time PCR was performed with the Eppendorf Mastercycler Realplex4 system. Custom oligos for mouse p53 (Forward: GCCATGGCCATCTACAAGAA, Reverse: ATCGGAGCAGCGCTCATG) and mouse *ESPL1* (Forward: GACCGTGACATTGACCGTTA, Reverse: TAGGCCGTAGGCTACAGGTG) were used for amplification. GAPDH (Forward: TGCACCACCAACTGCTTAGC, Reverse: GGCATGGACTGTGGTCATGAG) was selected for normalization.

### Spectral Karyotyping (SKY)

The cocktail of mouse chromosome paints was obtained from Applied Spectral Imaging (ASI, Vista, CA). Hybridization and detection were carried out according to the manufacturer's protocol, with slight modifications. Chromosomes were counterstained with DAPI. Images were acquired with a SD300H Spectra cube (ASI) mounted on a Zeiss Axioplan II microscope using a custom designed optical filter (SKY-1) (Chroma Technology, Brattleboro, VT), and analyzed using SKY View 2.1.1 software (ASI, Vista, CA) [59].

### Statistical Analysis

For the histological analysis a ratio of positively stained cell counts versus total cell counts was computed for each treatment and each genotype. The staining ratios within each genotype may not be independent. As such, an average of ratios was computed for each mouse genotype. The statistical significance of differences in mean genotype ratios between treatments were evaluated using two-tailed unequal variance Student's t-tests.

## Supporting Information

Figure S1 ***ESPL1^+/hyp^, p53^−/−^*** **mice show increased proliferation in normal splenocytes isolated from 3 month old mice.** Primary splenocytes isolated from WT, *p53^−/−^* and *ESPL1^+/hyp^, p53^−/−^* mice were analyzed for cellular proliferation. Proliferation levels are indicated by the percentage of cells in any cycling phase of the cell cycle (G1, S, G2, M) (**B**) calculated by counting Ki67 cells (red) as a percentage of total cells (DAPI, blue). (n = at least two mice for each genotype were used and for each genotype 5 microscope fields, 60X magnification and>500 cells were counted). The average counts for both mice in each genotype were plotted. Significant difference was observed in proliferation levels in the *ESPL1^+/hyp^; p53^−/−^* compared to all other cohorts (*P = 0.01*).
(TIF)

Figure S2 **Western blot analysis of endogenous Separase protein from testis derived from the** ***ESPL1^+/hyp^*** **mice in p53 heterozygous and homozygous backgrounds show lower level of Separase compared to wild type mice.** Western blot from testis tissue of mutant mice and their wild type littermate controls (as indicated by the genotypes) is shown (top). Densitometric quantification of Separase expression in the *ESPL1^+/hyp^* mice in p53 heterozygous and homozygous backgrounds compared to wild type after normalization to the expression of a housekeeping gene γ tubulin to compensate for loading control (bottom).
(TIF)
