# Supplementary figures and images for: Separase Loss of Function Cooperates with the Loss of p53 in the Initiation and Progression of T- and B-Cell Lymphoma, Leukemia and Aneuploidy in Mice
Source: PLoS One. 2011 Jul 25;6(7):e22167. doi: 10.1371/journal.pone.0022167 (PMC3143119; doi:10.1371/journal.pone.0022167)

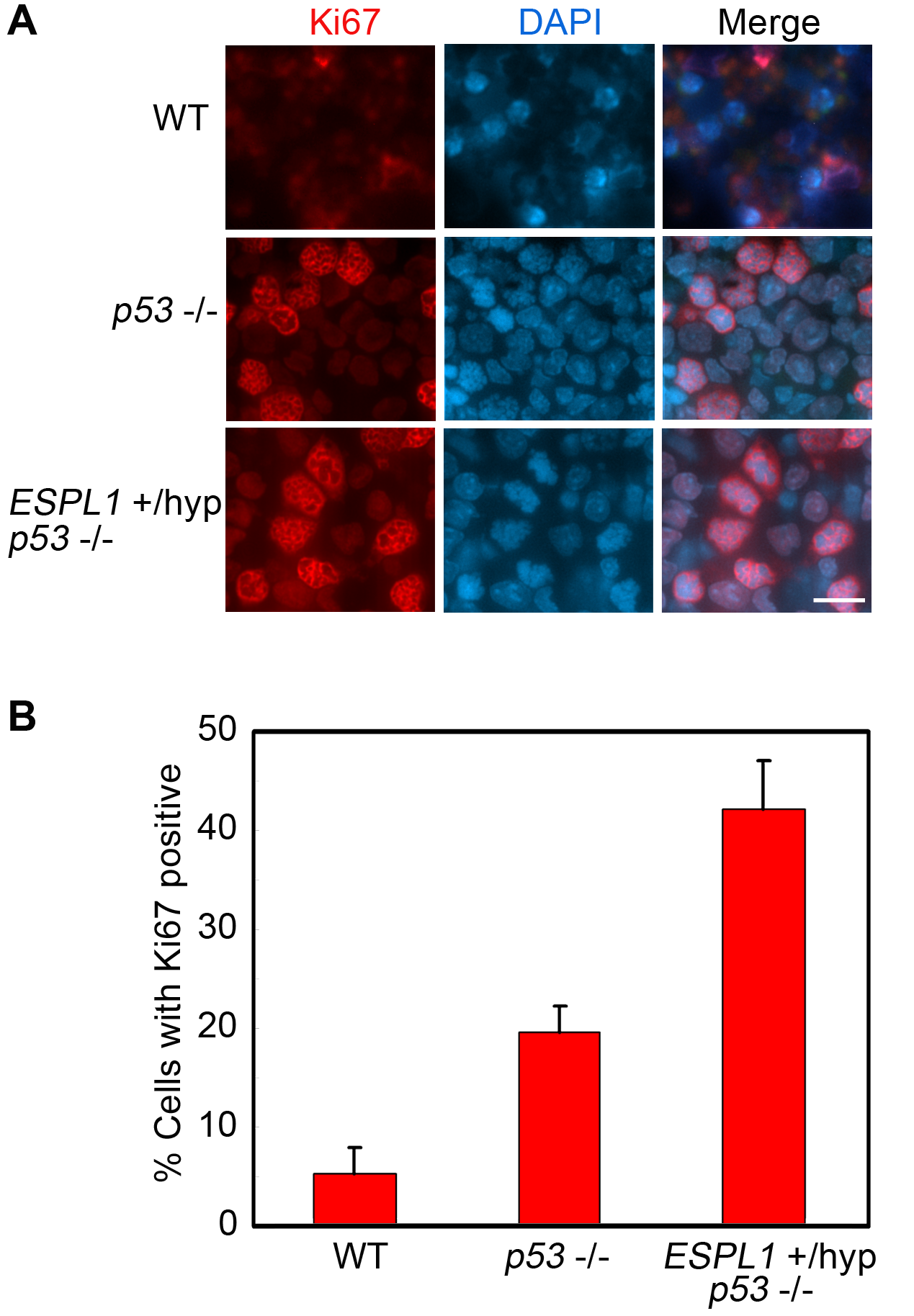

Supplement: Figure S1 — ESPL1+/hyp, p53−/− mice show increased proliferation in normal splenocytes isolated from 3 month old mice. Primary splenocytes isolated from WT, p53−/− and ESPL1+/hyp, p53−/− mice were analyzed for cellular proliferation. Proliferation levels are indicated by the percentage of cells in any cycling phase of the cell cycle (G1, S, G2, M) (B) calculated by counting Ki67 cells (red) as a percentage of total cells (DAPI, blue). (n = at least two mice for each genotype were used and for each genotype 5 microscope fields, 60X magnification and>500 cells were counted). The average counts for both mice in each genotype were plotted. Significant difference was observed in proliferation levels in the ESPL1+/hyp; p53−/− compared to all other cohorts (P = 0.01). (TIF) [file pone.0022167.s001.tif]

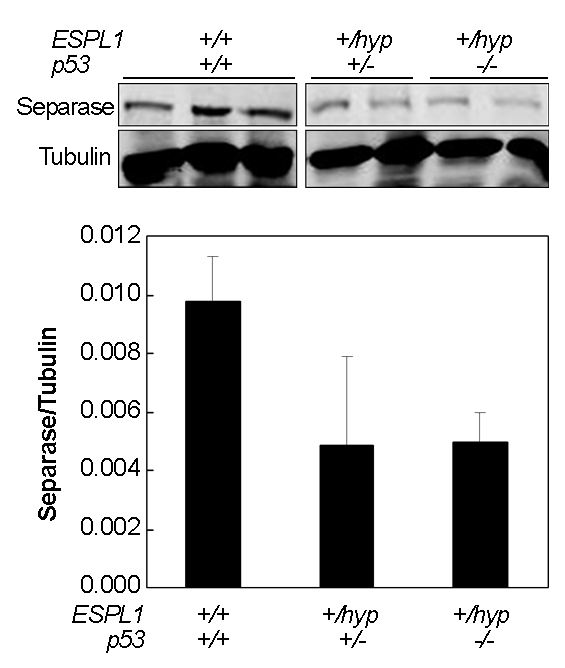

Supplement: Figure S2 — Western blot analysis of endogenous Separase protein from testis derived from the ESPL1+/hyp mice in p53 heterozygous and homozygous backgrounds show lower level of Separase compared to wild type mice. Western blot from testis tissue of mutant mice and their wild type littermate controls (as indicated by the genotypes) is shown (top). Densitometric quantification of Separase expression in the ESPL1+/hyp mice in p53 heterozygous and homozygous backgrounds compared to wild type after normalization to the expression of a housekeeping gene γ tubulin to compensate for loading control (bottom). (TIF) [file pone.0022167.s002.tif]
